# Signs for Girls: Biomarkers of Common Exposures

**Published:** 2007-01

**Authors:** Victoria McGovern

Urinary biomarkers are useful measures of environmental agents in the body. However, little is known about levels of such biomarkers in children and how they may vary by race, age, body mass index, and sex. A three-city pilot study reported this month used urinary biomarkers to better characterize a number of exposures in young girls **[*EHP* 115:116–121; Wolff et al.]**. The discovery of detectable urine levels of a range of hormonally active substances in the children may shed light on how various biomarkers relate to pubertal development.

The study authors measured parent compounds and metabolites of phytoestrogens, phthalates, and phenols in the urine of girls aged 6 to 9. They tested for 25 biomarkers from 22 agents including triclosan (an antimicrobial agent found in many household products), enterolactone (a micronutrient from seeds and grains), and monoethyl phthalate (a metabolite of phthalates used in shampoos, soaps, and cosmetics). The chemical classes studied were chosen because of their suspected hormonal activity and because they have been widely detected in the general population.

The 90 girls in the pilot study were recruited in New York, Cincinnati, and San Francisco, and included members of four racial/ethnic groups. The pilot study sampled a relatively small population to determine whether urinary biomarkers of the chemicals of concern would be detectable and variable enough for meaningful comparisons of their concentrations in relation to outcomes of female growth and development.

Most of the markers analyzed were found in more than 94% of the participants. Nine of them—including metabolites of isoflavones found in foods containing soy and of di(2-ethylhexyl) phthalate, a softening agent used in plastics—were found in all of the girls.

The team established that urinary metabolites of phenols, phthalates, and phytoestrogens in children are detectable and variable enough to make meaningful comparisons. And though the number of subjects was small, preliminary results point to variations in concentrations of some metabolites between girls of different races, as well as variation according to body mass index, and seasonal variation during the year—all factors that will be useful in dissecting the roles of the studied chemical classes in breast cancer and other diseases.

## Figures and Tables

**Figure:**
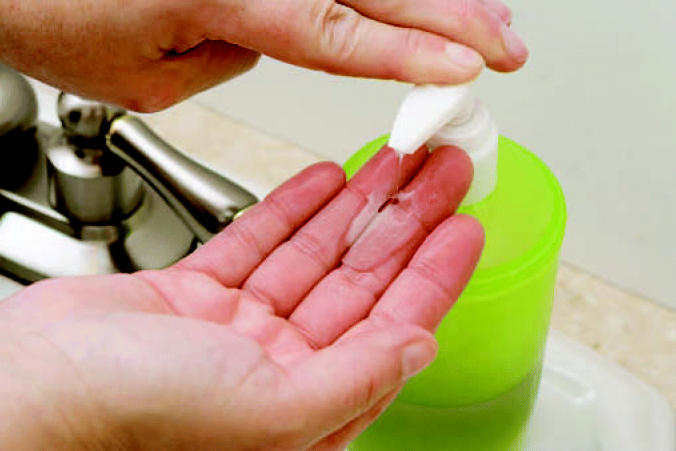
Chemicals in kids. Scientists have identified useful biomarkers of girls’ exposure to chemicals including agents found in antimicrobial soaps, shampoos, and other personal products.

